# Research Trends in Molecular Biological Studies on Oral Squamous Cell Carcinoma: A Bibliometric Analysis

**DOI:** 10.3389/or.2023.11585

**Published:** 2023-10-25

**Authors:** Indrayadi Gunardi, Irna Sufiawati, Hanna Goenawan, Dewi Marhaeni Diah Herawati, Ronny Lesmana, Ade Gafar Abdullah

**Affiliations:** ^1^ Faculty of Medicine, Universitas Padjadjaran, Bandung, Indonesia; ^2^ Oral Medicine Department, Faculty of Dentistry, Universitas Trisakti, Jakarta, Indonesia; ^3^ Oral Medicine Department, Faculty of Dentistry, Universitas Padjadjaran, Bandung, Indonesia; ^4^ Department of Biomedical Sciences, Faculty of Medicine, Universitas Padjadjaran, Bandung, Indonesia; ^5^ Central Laboratory, Universitas Padjadjaran, Bandung, Indonesia; ^6^ Department of Public Health, Faculty of Medicine, Universitas Padjadjaran, Bandung, Indonesia; ^7^ Department of Biomedical Sciences, Faculty of Medicine, Universitas Padjadjaran, Bandung, Indonesia; ^8^ Division of Biological Activity, Central Laboratory, Universitas Padjadjaran, Bandung, Indonesia; ^9^ Center of Excellence in Higher Education for Pharmaceutical Care Innovation, Universitas Padjadjaran, Bandung, Indonesia; ^10^ Electrical Engineering Studies Program, Universitas Pendidikan Indonesia, Bandung, Indonesia

**Keywords:** molecular biology, oral squamous cell carcinoma, bibliometric, oral cancer, OSCC

## Abstract

**Background:** Since the discovery of PCR and ELISA, *in vitro* research in the realm of molecular biology pertaining to oral squamous cell carcinoma (OSCC) has witnessed significant expansion.

**Objective:** to provide a comprehensive overview of molecular biology research on OSCC through visual mapping techniques.

**Methods:** We conducted an analysis of publications within the “oral squamous cell carcinoma” category from Scopus’ core collection. On 20 January 2023, we screened these publications using an advanced search employing the keywords “oral squamous cell cancer” and “cell line.” Data analysis was performed using Microsoft Excel 2010 and VOSviewer, facilitating the examination of author contributions, journal productivity, institutional affiliations, and contributions by nations. VOSviewer was further utilized for co-occurrence and reference analysis of keywords.

**Results:** A total of 781 papers spanning from 1992 to 2023 were collected. Notably, Japan, China, and the United States emerged as significant contributors in this field. The Osaka University Graduate School of Dentistry (Japan) ranked first with 21 publications. Chae J-I of Chonbuk National University (South Korea) emerged as the most prolific author, with 14 publications. The International Journal of Oncology and the Journal of Oral Pathology and Medicine were identified as the two most prolific journals. The central themes that emerged were epidermal growth factor receptor, invasion, epithelial-mesenchymal transition, angiogenesis, apoptosis, and metastasis.

**Conclusion:** The rate of publications focused on the molecular biology of OSCC has seen a remarkable increase. Research priorities have shifted from topics such as “radiation, RANKL, cyclin D1, RNA interference, and matrix metalloproteinase” to encompass areas such as “chemoresistance due to cisplatin, other therapeutic agents (metformin and monoclonal antibody), autophagy, inflammation, microRNA, cancer-associated fibroblasts, and STAT3 (with roles in cell migration and tumorigenesis).” These seven significant future research areas hold promise in identifying reliable biological markers for oral cancer detection and treatment, thereby improving clinical outcomes.

## Introduction

Oral squamous cell carcinoma (OSCC) accounts for approximately 4% of all malignant tumors globally, with 300,000 new occurrences each year [1]. Surgical, radiotherapeutic, and chemotherapeutic approaches have been used to treat OSCC. Previous research found that five- and 10-year survival rates were 39%–42% and 38%, respectively, and that tumor sites were associated with a favorable prognosis [2, 3]. In addition, numerous tumor types of OSCC cell lines have been examined in biomolecular research on distinct genetic backgrounds, including gender, ethnicity, tumor site, presence of metastases, primary or secondary tumors, and viral involvement. Consequently, biomarker patterns have been found to differ [4]. However, numerous molecular genes are tightly connected, polarizing in a cascade of migration, proliferation, apoptosis, and metastasis, which makes it difficult to understand the OSCC mechanism.

A deeper understanding of the molecular factors involved in OSCC pathogenesis is critically important. Insights into the biological mechanisms that promote OSCC development and progression can help identify reliable biomarkers for early detection, as well as novel therapeutic targets. Molecular research may also shed light on the heterogeneity observed in OSCC tumors, as well as differences in racial, ethnic, and geographic distributions.

Scientometric analysis and text mining are generally acknowledged methods for examining substantial amounts of data in a certain sector [5]. Bibliometric techniques are typically used to assess and depict publishing trends, prolific authors, central themes, and research frontiers within a specific area. In 2019, only the etiology and risk factors in the most frequently cited articles on squamous cell carcinoma of the mouth, lips, and oropharynx were explored [6]. Several studies on the bibliometric analysis of squamous cell carcinoma have been published since 2019. In 2021, two publications investigated nanotherapy for HNSCC [7] and neck dissection for OSCC [8]. In 2022, analyses of OSCC risk factors [9] and correlations between macrophages and oral cancer were published [10]. However, a comprehensive synthesis of the accumulated biomolecular findings on OSCC is lacking. No previous study has discussed biomolecular research on OSCC or any other research area. Therefore, this study aimed to map the research landscape in this field to date, elucidating key trends and knowledge clusters that could inform future research priorities and strategies. Further molecular biological research on OSCC is urgently needed to improve patient outcomes. Because numerous biomolecular OSCC research studies have been undertaken, the present study utilized VOSviewer to produce a visualization of previous studies on OSCC.

### Research Questions

RQ1 What are the key growth trends in the literature on molecular biological studies of OSCC?

RQ2 What authors, institutions, journals, documents, and countries from emerging regions have had the greatest influence on molecular biological studies on OSCC?

RQ3 What are the intellectual structures of molecular biological studies on OSCC?

RQ4 What topics in the molecular biological literature on OSCC have been studied with the greatest frequency and have attracted the greatest amount of attention?

## Methods

### Search Strategy

Because of the extensive utilization of cell lines in molecular biological studies on OSCC and head and neck squamous cell carcinoma, it may be difficult to distinguish the origin of cells derived specifically from OSCC. Thus, previous studies on head and neck squamous cell carcinoma, metastasis, and unknown cell line tumor types were excluded. In this study, the Scopus database was used to meet the requirements of VOSviewer. The inclusion criteria for abstracts of articles on the OSCC cell line were defined. On 20 January 2023, we performed an advanced search using the following criteria:

ABS(“oral squamous cell carcinoma” OR oscc) AND ABS(A253 OR BB49HNC OR BHY OR BICR10 OR BICR16 OR BICR22 OR BICR31 OR BICR56 OR BICR78 OR CA922 OR CAL27 OR CAL33 OR H103 OR H157 OR H357 OR H376 OR H413 OR “HN” OR HO1N1 OR HO1U1 OR HSC2 OR HSC3 OR HSC4 OR KON OR KOSC2 OR OCC-22 OR OCC-25 OR OCC-31 OR OCC-32 OR OCC-34 OR ORL-115 OR ORL-136 OR ORL-150 OR ORL-174 OR ORL-188 OR ORL-204 OR ORL-48 OR OSC19 OR OSC20 OR PCI30 OR PCI38 OR PCI6A OR PECAPJ15 OR PECAPJ34CLONEC12 OR PECAPJ41CLONED2 OR PECAPJ49 OR SAS OR SCC15 OR SCC25 OR SCC4 OR SCC9 OR SKN3 OR TR146 OR UPCISCC026 OR UPCISCC040 OR UPCISCC074 OR UPCISCC090 OR UPCISCC111 OR UPCISCC116 OR UPCISCC131 OR UPCISCC152 OR UPCISCC154 OR UPCISCC200 OR YD10B OR YD15 OR YD8) AND (LIMIT-TO (SRCTYPE,“j”)) AND (LIMIT-TO (DOCTYPE,“ar”)) AND (LIMIT-TO (LANGUAGE,“English”)).

### Data Extraction and Bibliometric Analysis

**FIGURE 1:**
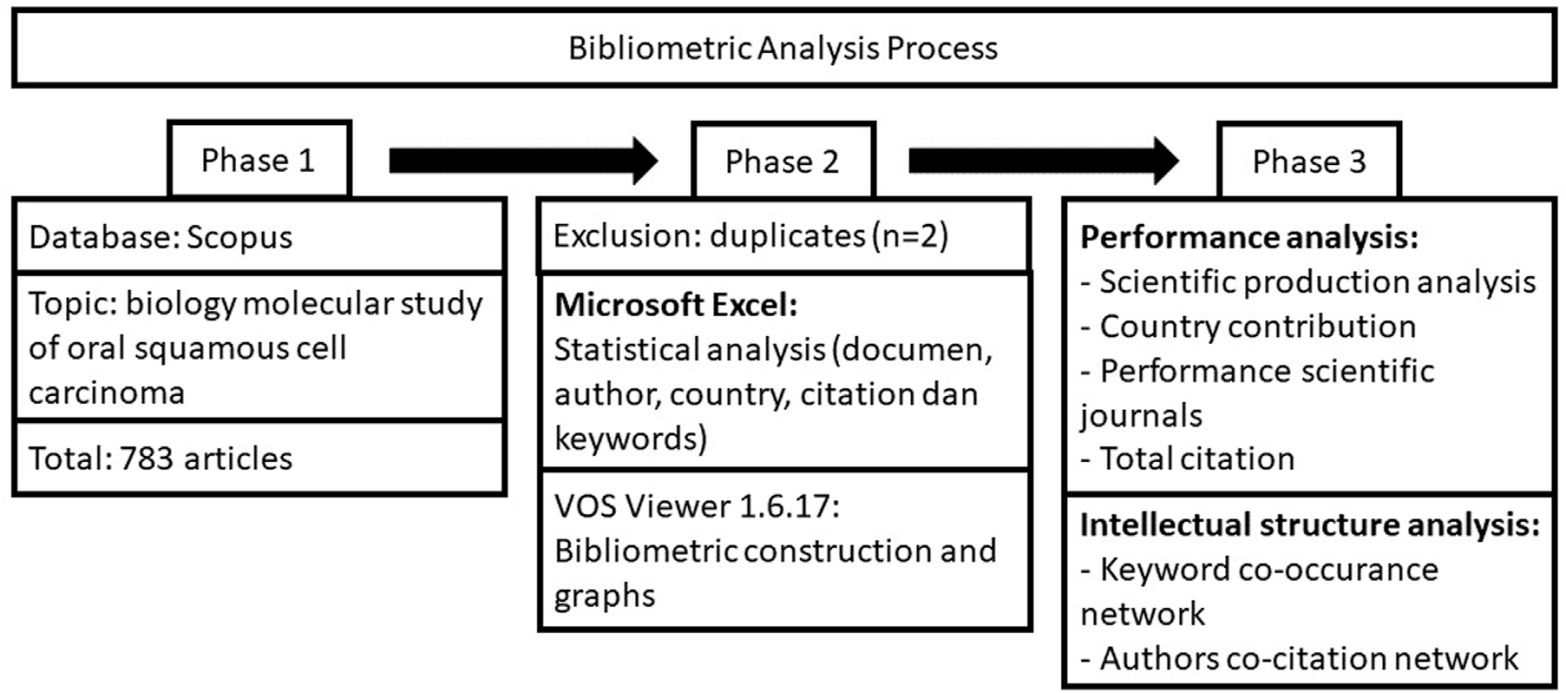
Flow diagram of inclusion process.

To determine who contributed the most, the bibliometric parameters (i.e., title, keywords, journal, publication year, citations, author, institution, and country) were extracted and imported into Microsoft Excel 2010 (Redmond, Washington, United States) and VOSviewer 1.6.17 (Leiden University, Leiden, The Netherlands) [11]. In VOSviewer, node size is positively related to the number of articles. The co-authorship study assessed the collaboration between various countries, authors, and institutions (Figure 1). Cooperation strength was positively correlated with the total link strength between the two nodes. Using VOSviewer, the co-occurrence and reference analysis keywords were visualized. An independent investigator examined the data extraction for any bias in the scientometric analysis in three phases.

## Results

### General Data

**FIGURE 2:**
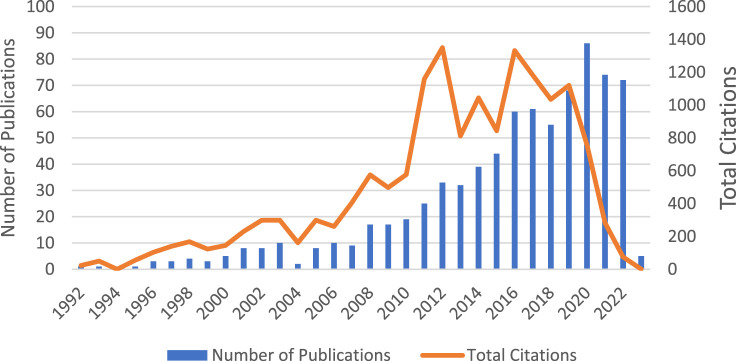
Total number of publications and citations based on year.

A total of 783 articles (original research) published from 1992 to 2023 were extracted for analysis. Two articles were excluded because they were duplications. Trends in the number of published articles are displayed in Figure 2. As shown in Figure 2, fewer than 10 articles were published in the first decade. The highest number of articles (*n* = 86) was published in 2020. The highest numbers of citations were in 2012 and 2016 (1,331 and 1,350, respectively). The number of citations per article was 19.67. The total number of citations was 15,406. A total of 289 journals published these articles.

### Author Collaborations, Institutions, and Journal Analysis

**FIGURE 3:**
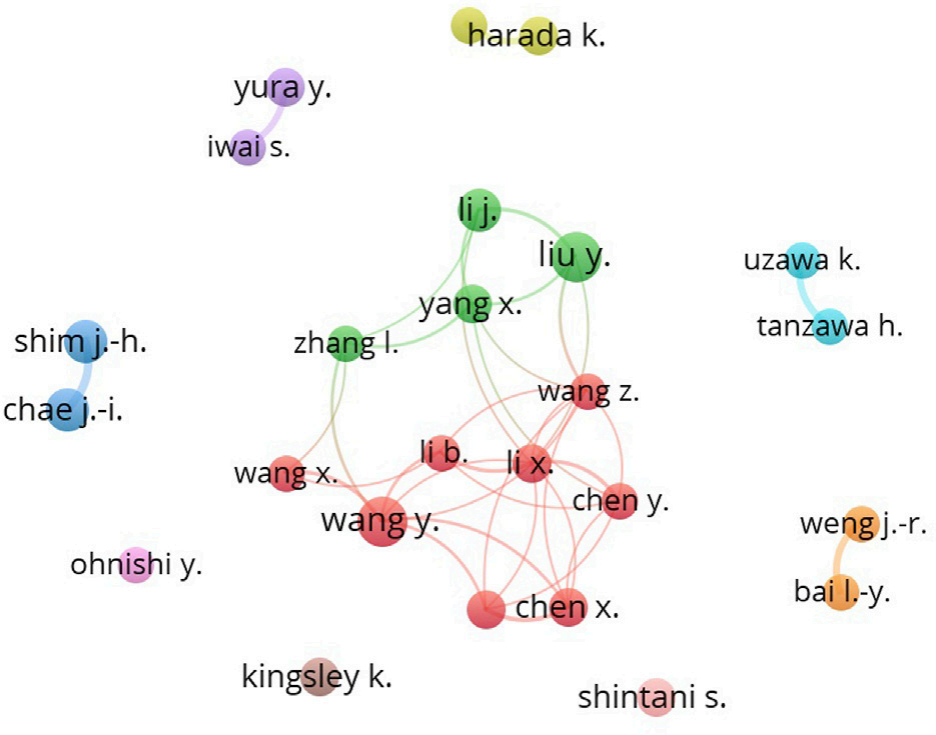
Intellectual structure based on co-authorship.

Figure 3 shows 10 clusters of author collaborations in molecular biological studies on OSCC. This visualization was obtained by a minimum of 10 articles by each author; thus, only 25 authors were included. Only 12 authors showed strong links in the network. Table 1 presents a list of prolific authors, in which Chae, Jung-Il had the highest number of citations per publication at 19.35. Chae, Jung-Il, and Shim, Jung-Hyun published 14 articles that included 271 citations. Regarding the institution, the Osaka University Graduate School of Dentistry (Japan) and the China Medical University (Taiwan) had the highest number of publications.

**TABLE 1:** Top 10 most prolific authors.

Rank	Author	Number of publication	TC	CPP	Institution	Country
1	Chae, Jung-Il	14	271	19.35	Chonbuk National University	South Korea
2	Shim, Jung-Hyun	14	271	19.35	Mokpo National University	South Korea
3	Shintani, Satoru	11	329	29.90	Showa University	Japan
4	Yura, Yoshiaki	11	199	18.09	Osaka University Graduate School of Dentistry	Japan
5	Kingsley, Karl	11	149	13.54	University of Nevada	United States
6	Iwai, Soichi	10	252	25.2	Osaka University Graduate School of Dentistry	Japan
7	Chang, Kuo-Wei	10	253	25.3	National Yang-Ming University	Taiwan
8	Bai, Li-Yuan	10	207	20.7	China Medical University	Taiwan
9	Weng, Jing-Ru	10	207	20.7	China Medical University	Taiwan
10	Uzawa, Katsuhiro	10	195	19.5	Chiba University	Japan

TC, total citations; CPP, citation per publication.

Table 2 shows the top 10 journals that published research on the molecular biology of OSCC. These journals were categorized in the first and second quartiles based on SCImago journal rankings. The *Journal of Oral* Pathology and Medicine published 42 articles but had a low number of citations per publication score (14.38) compared with the *International Journal of Oncology*, which had 35 articles but the highest number of citations per publication (28.62). Only one journal, *Oncology Letters*, was ranked in the third quartile.

**TABLE 2:** Top 10 most frequently cited journals.

Rank	Journal	Publication	TC	CPP	SNIP^a^	SJR^b^
1	Journal of Oral Pathology and Medicine	42	604	14.38	0.370	0.77 Q1
2	International Journal of Oncology	35	1002	28.62	1.125	1.11 Q2
3	Oncology Reports	35	533	15.22	0.786	0.81 Q2
4	Oral Oncology	33	832	25.21	1.85	1.42 Q1
5	International Journal of Molecular Sciences	18	242	13.44	1.401	1.18 Q1
6	Oncology Letters	18	257	14.27	0.725	0.64 Q3
7	PLoS ONE	18	594	33	1.368	0.85 Q1
8	Frontiers in Oncology	17	111	6.52	1.191	1.29 Q1
9	Oncotarget	15	269	17.93	1.019	0.97 Q2
10	Anticancer Research	12	137	11.41	0.721	0.6 Q2

TC, total citations; CPP, citation per publication; SJR = SCImago journal Rank.

^a^ 2023 provided by SCOPUS.

^b^ 2023 provided by ScimagoJR.

### Document Citation Analysis

Table 3 shows the highest number of documents cited. Only 10 articles were cited in more than 100 documents. The most frequently cited author was Arti Yadav at the Ohio State University Comprehensive Cancer Center, who published 12 articles that were cited in 1,200 documents.

**TABLE 3:** Top 10 most frequently cited publications.

Rank first author	Article title	Journal	Document type	TC
[13]	IL-6 promotes head and neck tumor metastasis by inducing epithelial-mesenchymal transition via the JAK-STAT3-SNAIL signaling pathway	Molecular Cancer Research	Article	371
[14]	Humanized anti-interleukin-6 receptor antibody suppresses tumor angiogenesis and *in vivo* growth of human oral squamous cell carcinoma	Clinical Cancer Research	Article	137
[15]	Elevated Bmi-1 expression is associated with dysplastic cell transformation during oral carcinogenesis and is required for cancer cell replication and survival	British Journal of Cancer	Article	132
[16]	Porphyromonas gingivalis promotes invasion of oral squamous cell carcinoma through induction of proMMP9 and its activation	Cellular Microbiology	Article	131
[17]	Nonadhesive culture system as a model of rapid sphere formation with cancer stem cell properties	PLoS ONE	Article	127
[18]	C-terminal domain of human CAP18 antimicrobial peptide induces apoptosis in oral squamous cell carcinoma SAS-H1 cells	Cancer Letters	Article	117
[19]	Stress hormones increase cell proliferation and regulates interleukin-6 secretion in human oral squamous cell carcinoma cells	Brain, Behavior, and Immunity	Article	113
[20]	Tumor-associated macrophages correlate with the clinicopathological features and poor outcomes via inducing epithelial to mesenchymal transition in oral squamous cell carcinoma	Journal of Experimental and Clinical Cancer Research	Article	110
[21]	Increased expression of lncRNA CASC9 promotes tumor progression by suppressing autophagy-mediated cell apoptosis via the AKT/mTOR pathway in oral squamous cell carcinoma	Cell Death and Disease	Article	108
[22]	Expression of integrin β6 enhances invasive behavior in oral squamous cell carcinoma	Matrix Biology	Article	107

TC, total citations.

### Country Citations Analysis

**FIGURE 4:**
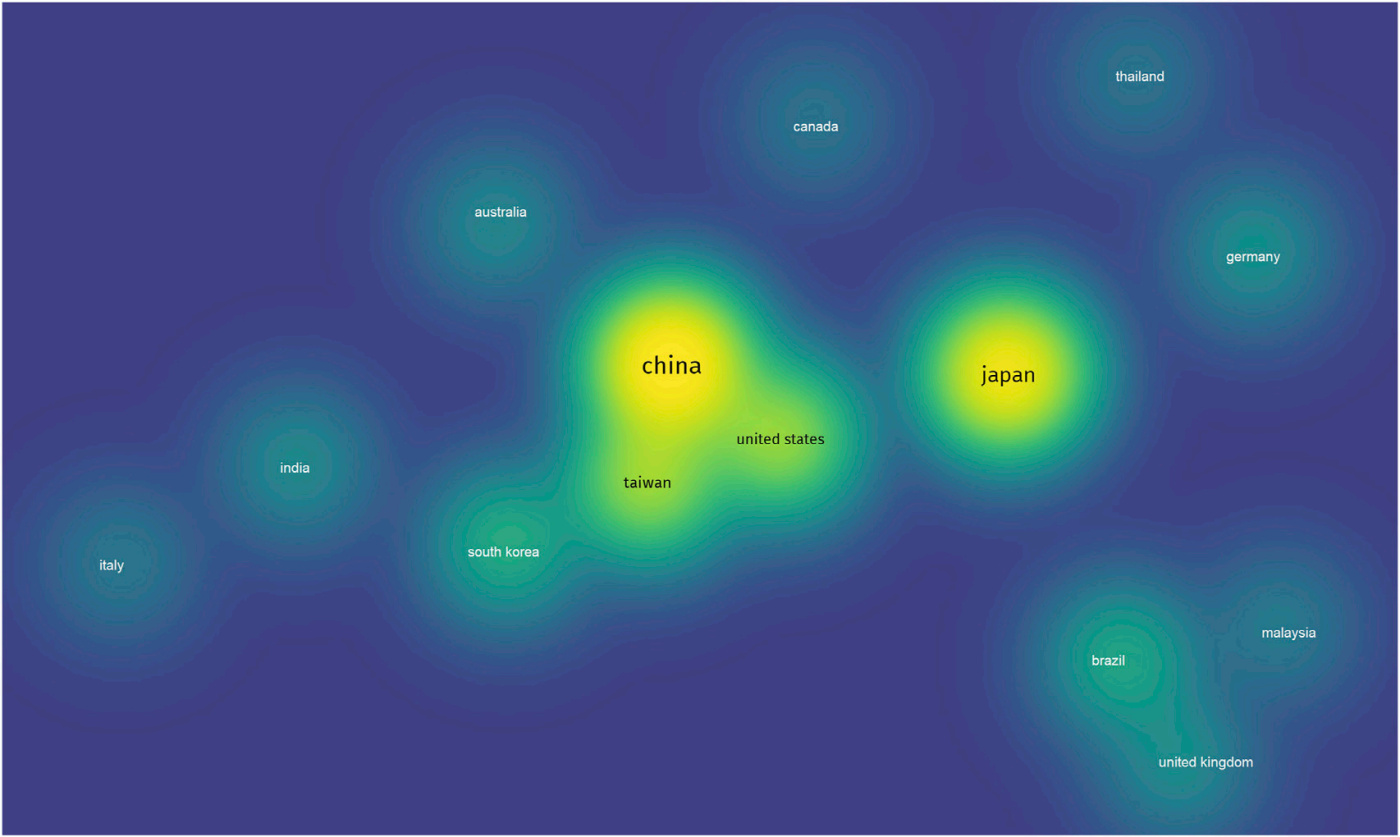
Visual density of citations based on country.

The results of the analysis of countries that were the most frequently cited are shown in Figure 4. Japan, which has 210 articles, was cited in 4,133 documents, followed by China, which had 265 articles cited in 3,730 documents. The United States had 101 articles cited in 3,102 documents, and Taiwan had 105 articles cited in 2,610 documents. Other countries had fewer than 40 articles that were cited in less than 750 documents. These results were supported by several researchers in Japan (Table 1).

### Intellectual Structure of Author Keywords

**FIGURE 5:**
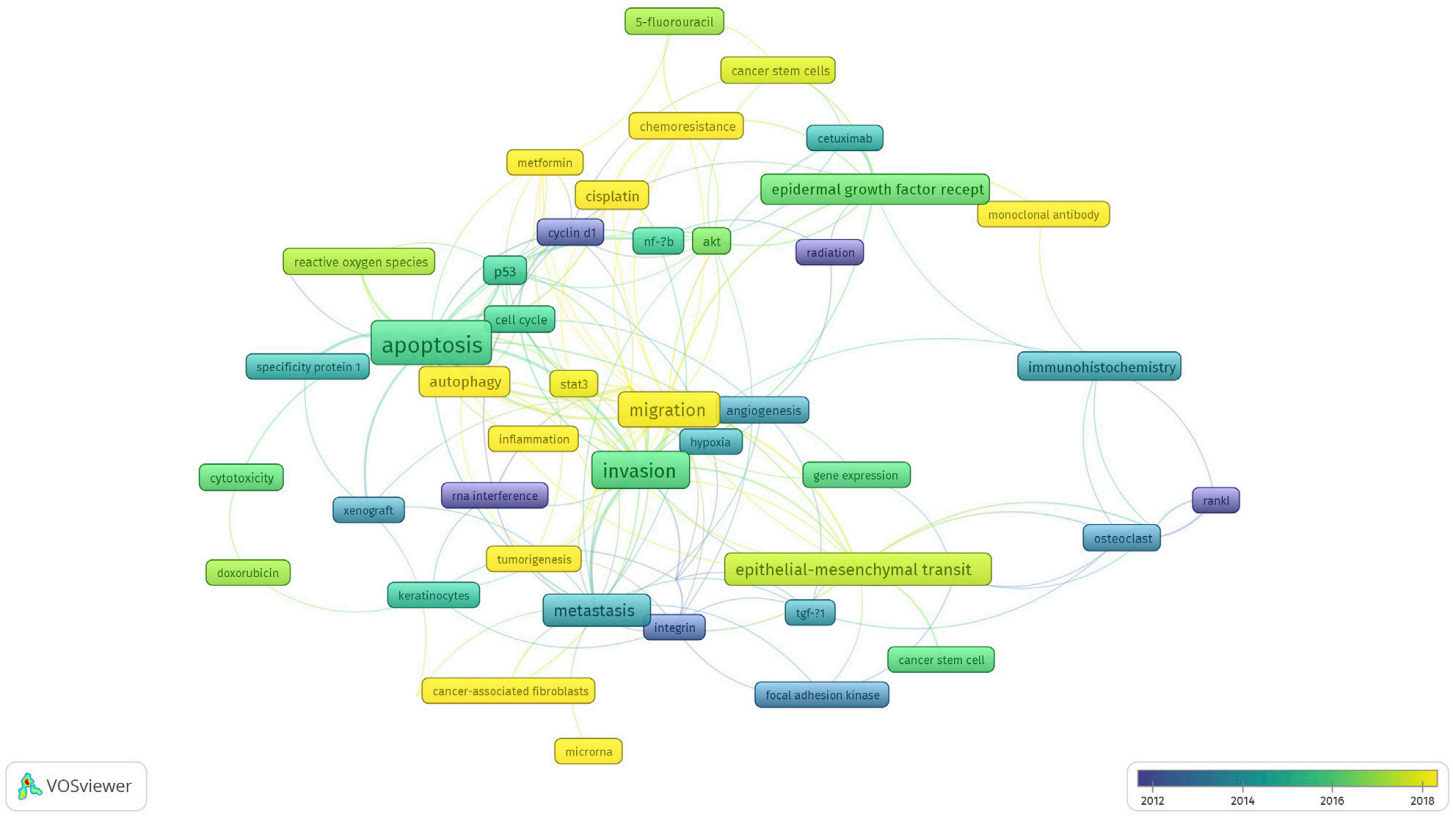
Topics in molecular biological studies on OSCC.

Figure 5 illustrates the subject groups and temporal trends of topic keywords, which were determined by analyzing data on the research publications.

## Discussion

In the field of molecular biology, there has been increasing interest in the study of OSCC, particularly in the last decade. This can be attributed to the increasing number of patients diagnosed with this cancer and the number of cases in which tumors exhibit resistance to medical treatment.

Numerous studies on OSCC have been undertaken. Since the invention of PCR and ELISA techniques, the amount of research on this subject has increased dramatically. Moreover, an increasing number of molecular biological studies on OSCC have been conducted with the goal of overcoming oral cancer. The dissemination of previous results has been necessary for a further theoretical and applicable understanding of the molecular mechanism of the development of oral cancer [12].

Regarding molecular biological research on OSCC, Figure 3 provides a visual representation of 10 distinct clusters denoting author collaborations. Among the 25 authors who met the minimum document requirement of 10, only 12 authors demonstrated significant network connections. As shown in Table 1, Chae, Jung-Il emerged as the most productive author, contributing 14 articles that generated a total of 271 citations. Notably, Chae, Jung-Il also achieved the highest number of citations per publication score of 19.35. Shim, Jung-Hyun published a total of 14 articles that were cited 271 times. Regarding institutional rankings, Table 2 shows that the Osaka University Graduate School of Dentistry in Japan ranked the highest with 21 publications, followed by China Medical University in Taiwan with 17 publications. The majority of the top 10 journals were ranked in the first and second quartiles, according to SCImago journal rankings. Notably, the *International Journal of Oncology* led with 35 publications and the highest citation per publication score of 28.62. The third quartile consisted of a single journal, *Oncology Letters*.

As shown in Table 3, 10 articles were cited more than 100 times. Notably, Arti Yadav, who is affiliated with the Ohio State University Comprehensive Cancer Center, emerged as the leading author with 12 publications and 1,200 citations. The data presented in Figure 4 and Table 1 show the distribution of citations by country. Japan led with 210 publications, which were cited a total of 4,133 times, followed by China, with 265 articles and 3,730 citations. The United States ranked third with 101 articles and 3,102 citations, and Taiwan ranked fourth with 105 articles and 2,610 citations. The remaining countries contributed fewer than 40 articles, each of which received fewer than 750 citations. These results were consistent with the high representation of Japanese researchers, as shown in Table 1.

Keywords were extracted automatically to develop network co-occurrence based on bibliographies. Keywords were counted using the full counting method. To avoid repeated terms, evaluations and revisions were conducted manually to develop a custom thesaurus. In this data analysis, author keywords were set at a minimum number of occurrences of five, and 52 articles met this criterion. The network presented in Figure 5 included several nodes that reflected keywords and edges that were correlated. The distance between nodes indicated the strength or weakness of a correlation. Similar keywords were categorized into clusters.

The following topics on the molecular biology of OSCC were studied with the greatest frequency: apoptosis, cell invasion, cell migration, tumor proliferation, and metastasis. Compared to other cancers, the pathogenesis of oral cancer has not been clearly understood until now. The biological marker that plays a role in triggering the transformation cascade may differ from that in a population study [2, 3]. Early research focused on several nodes, such as radiation, RANKL, cyclin d1, RNA interference, and matrix metalloproteinase. However, the topic of chemoresistance due to cisplatin, other therapeutic agents (metformin and monoclonal antibody), autophagy, inflammation, microRNA, cancer-associated fibroblasts, and STAT3, which play a role in cell migration and tumorigenesis, have recently attracted the greatest amount of attention. The modality treatment of cancer is to inhibit its proliferation (i.e., cancer-associated fibroblasts) by using chemotherapeutic or other therapeutic agents. However, in reality, chemoresistance is due to STAT3 [23, 24].

Based on the titles of the articles, the findings of our study showed six clusters of research: epidermal growth factor receptor, invasion, epithelial–mesenchymal transition, angiogenesis, apoptosis, and metastasis. The total number of links obtained was 49, with a total link strength of 664 (Figure 5).

Cluster 1 was an epidermal growth factor receptor with the keywords cisplatin, chemoresistance, Akt, NF-kb, 5-fluorouracil, cancer stem cells, cetuximab, monoclonal antibody, and radiation. Cluster 2 was invasion with the keywords migration, proliferation, cell cycle, cyclin d1, gene expression, inflammation, metformin, and RNA interference. Cluster 3 was the epithelial–mesenchymal transition with the keywords immunohistochemistry, osteoclast, bone invasion, cancer stem cell, e-cadherin, focal adhesion kinase, and RANKL. Cluster 4 was angiogenesis with the keywords cytotoxicity, matrix metalloproteinase, integrin, doxorubicin, hypoxia, keratinocytes, and TGF-B1. Cluster 5 was apoptosis with the keywords autophagy, p53, reactive oxygen species, stat3, specificity protein 1, photodynamic therapy, and xenograft. Cluster 6 was metastasis with the keywords prognosis, tumorigenesis, cancer-associated fibroblasts, microRNA, and tumor microenvironment.

The limitation of this study was its use of only the Scopus database. Future biometric studies should include other databases, such as the Web of Science and Dimensions AI. Moreover, gray journals should be carefully evaluated.

## Conclusion

The bibliometric analysis performed in this study provides information about research on the molecular biology of OSCC published between 1992 and 2023. The results showed six clusters of research: epidermal growth factor receptor, invasion, epithelial–mesenchymal transition, angiogenesis, apoptosis, and metastasis. Based on these findings, further research on OSCC should focus on the therapeutic treatment of oral cancer. Overall, there has been a significant increase in research on oral cancer, particularly in the last decade. This may reflect the increasing number of cases of OSCC in the population and the potential for tumor resistance against chemotherapeutic agents.

## Data Availability

The original contributions presented in the study are included in the article/supplementary material, further inquiries can be directed to the corresponding author.
